# Call for Papers—Journal of Epidemiology Reprints of Pioneering Papers Series: Spotlighting Little-Known Non-English Language Research Papers From Japan and Around the World

**DOI:** 10.2188/jea.JE20190262

**Published:** 2020-01-05

**Authors:** Naoki Kondo

**Affiliations:** Department of Health Education and Health Sociology, School of Public Health, the University of Tokyo, Tokyo, Japan

**Keywords:** pioneering studies, public health, policy implication

English has been and continues to be extensively used as a common language in much of the political discourse and argumentation that occurs worldwide, with the vast majority of scientific papers and academic conferences/meetings also using English. It follows, then, that academic papers written in other languages would surely be less well represented, having fewer opportunities to be read and evaluated globally. This may also link to various difficulties in the promotion of epidemiology and other scientific fields. In practice, an epidemiologist may consider publishing papers in English for several reasons: (1) The research deserves to be read by a global audience, as it is novel and has implications in other areas or has universally applicable findings and policy implications; (2) publishing papers in English is required to advance one’s career; or (3) the researcher is a native English speaker or someone who likes writing in English.

Every epidemiologist knows the heroic story of John Snow and the pioneering work he undertook to control the cholera outbreak that occurred in London in 1854. The fact that it happened in an English-speaking country would seem to be a major reason why this story has become famous globally, despite the events unfolding in a small district in downtown London. Or, to put it another way, if a modern-day epidemiologist comes from a non-English speaking area and sees no merit in any of the reasons listed above for publishing in English, then regardless of the potentially wider impact of the research, he/she may well choose to only write in his/her own native language, which would be a rational and reasonable choice. Writing in one’s own language should have clear advantages: low financial, mental, and time costs associated with writing, while having the potential to make an important contribution locally.

Despite this, in the current era of globalization, important epidemiologic data on any emerging disease, especially if it is contagious like Ebola, is likely to be published in English. However, the situation was very different in previous decades; that is, epidemiological research was much more of a local activity than it is today. Hence, it is highly likely that there are vast numbers of epidemiologic papers that are not written in English, but that, nevertheless, deserve to be published in English and read by a global audience given their potential contemporary relevance. Moreover, it is likely that much of this research may even exist from many years ago, given the almost total absence of scientists publishing epidemiological research in English until only recently in many countries across the world.

Some of these papers may have important public health implications even now. For example, early-stage epidemiologic surveys of a local disease outbreak may be especially valuable, particularly if the disease subsequently spreads to many areas of the world in later years. A good example of this comes from Japan, with epidemiological research on diseases related to industrial pollution, including Minamata disease (organic mercury poisoning), Itai-itai disease (cadmium intoxication), and Yokkaichi asthma (an asthma caused by industrial air pollution). Other potentially valuable papers are those that describe the history of how local disease was eradicated. For example, epidemiologic papers on the story of how *schistomatosis japonica* was eradicated in the Kofu basin, Yamanashi, Japan belong in this category: 100 years of multidimensional interventions, including ways to ensure a sanitary environment and behaviors, research on civil engineering, medication, community organizing, and policy initiatives are extraordinarily interesting and have many potential implications not only for epidemiology and public health but also for the fields of medicine, governmental policy, engineering, cultural science, equity, and justice. These materials could have a significant impact in terms of global health, especially as a case study in disease eradication. However, information written in English about such topics is still very limited.

With these thoughts in mind, I, as an editorial board member of the Journal of Epidemiology (JE), asked all the Associate Editors if they knew of “any papers, written in Japanese or other non-English languages that deserved to be translated into English, so that they could be read by a global audience”. Since that time, several candidate papers have been suggested. Subsequently, the editorial board of JE decided to launch a Reprints of Pioneering Papers series in order to spotlight these papers. In this series, we will publish reprints of these groundbreaking papers translated into English together with an invited commentary by an epidemiologist eminent in that specific field of research, providing a more comprehensive explanation of the topic and its implications for the current era.

In relation to this, we now invite readers of JE to nominate possible papers to be included in this series. Those who have potential papers in mind should contact the JE editorial office (edit@jeaweb.jp) via email, while providing a copy of the suggested paper and a brief explanation of why the paper is pioneering and has potentially important implications today (this text should not exceed 300 words). If the proposed paper is not written in Japanese and there is no supporting information in either Japanese or English (eg, an English abstract), please also attach a summary of the paper written in Japanese or English.

The first paper in this series is about Minamata disease, a disease caused by industrial pollution that emerged in the early stage of Japan’s post-war economic boom in the late 1950s. The outbreak of this disease, which is caused by chronic organic mercury poisoning, occurred in two bay areas: Minamata (Kumamoto Prefecture) and Niigata (Niigata Prefecture), resulting in 2,280 officially recognized fatalities. The legal dispute about what took place and its effects is still ongoing with the current discussion focusing on second-generation victims (those who were exposed in utero) and the discrimination being experienced by them. The seminal paper by Shoji Kitamura, who was Professor of Public Health at Kumamoto University at that time, presents statistical findings from his epidemiological research and draws a firm conclusion about the cause of the emerging disease: that it was poisoning due to eating polluted fish, despite the fact that, just like in John Snow’s time, no one, including Kitamura himself, knew that the disease was caused by organic mercury.^1^ Nonetheless, together with his colleagues, Kitamura ruled out various hypotheses on the cause of the disease that were suggested by the local authorities at that time, including that the disease was infectious, congenital, and caused by polluted drinking water or malnutrition.

I feel certain that the readers of JE will find this research and the story of it just as exciting and informative as reading about John Snow’s work for the first time, as the paper by Professor Kitamura and his coworkers together with Takashi Yorifuji’s accompanying commentary provide a unique window into a world of, as yet, little-known but potentially valuable research.^2^
